# The Clinical Impact of Dental Implant Placement in Close Proximity to Natural Teeth: A Systematic Review and Meta-Analysis

**DOI:** 10.7759/cureus.99077

**Published:** 2025-12-12

**Authors:** Nilesh V Joshi, Prajakta Rao, Prakash Talreja, Mridula Joshi, Anupa R Shetty, Sanpreet S Sachdev, Shifa Khan

**Affiliations:** 1 Department of Periodontology, Bharati Vidyapeeth (Deemed to be University) Dental College and Hospital, Navi Mumbai, IND; 2 Department of Prosthodontics and Crown and Bridge, Bharati Vidyapeeth (Deemed to be University) Dental College and Hospital, Navi Mumbai, IND; 3 Department of Oral Pathology and Microbiology, Bharati Vidyapeeth (Deemed to be University) Dental College and Hospital, Navi Mumbai, IND; 4 Department of Periodontics, Bharati Vidyapeeth (Deemed to be University) Dental College and Hospital, Navi Mumbai, IND

**Keywords:** dental implants, implant proximity, marginal bone loss, pulpal pathology, tooth injury

## Abstract

The spatial relationship between dental implants and adjacent natural teeth is critical for maintaining peri-implant health and avoiding iatrogenic complications. This systematic review and meta-analysis evaluated the biological and structural effects of implant placement within 1.5 mm of natural tooth roots or in direct contact with them. Electronic searches were performed in PubMed/MEDLINE, Cochrane Library, Scopus, Web of Science, ScienceDirect, Google Scholar, and Semantic Scholar databases from inception to January 2025, following Preferred Reporting Items for Systematic Reviews and Meta-Analyses 2020 guidelines (PROSPERO ID CRD42024499790). Eleven studies met the inclusion criteria, comprising eight observational studies and three case series, representing 1,727 participants and over 2,000 implants. Pulpal and periapical pathology were the most frequently reported adverse outcomes, with 20%-25% of affected teeth requiring root canal therapy in proximity-contact cases. Marginal bone resorption and peri-implantitis were observed in up to 4% of implants placed less than 1 mm from adjacent roots. Secondary caries and cervical structural defects were reported less consistently but were more prevalent in posterior regions. Meta-analysis using a random-effects model yielded a pooled effect size of 0.11 (95% confidence interval: 0.03-0.22), indicating a low yet clinically relevant risk of proximity-related complications, with an overall implant survival exceeding 95%. The certainty of evidence was moderate for pulpal and bone outcomes and low for caries or structural changes. Within the limitations of the available data, implants placed closer than 1.5 mm to natural teeth may cause localized biological damage without significantly compromising implant longevity. Meticulous presurgical planning and guided placement are essential to prevent avoidable injury.

## Introduction and background

The success of dental implant therapy depends not only on osseointegration but also on precise spatial placement that respects the surrounding anatomic and biological structures. Modern implantology strives to restore esthetics, function, and comfort through prosthetically driven positioning that mimics natural tooth morphology. However, clinical reality often presents constraints such as limited edentulous space, root angulations, or resorbed alveolar ridges, making ideal implant placement challenging [1]. In such conditions, the implant may inadvertently encroach upon the adjacent tooth, raising concerns about iatrogenic injury and long-term complications.

Current surgical guidelines recommend maintaining a minimum horizontal distance of 1.5 mm between an implant and the root surface of a neighboring natural tooth [2]. This safety margin preserves the vascular network of the interproximal bone and allows the formation of the supracrestal tissue complex, which is essential for peri-implant health. Deviation from this guideline can occur due to operator error, inadequate presurgical planning, or lack of three-dimensional imaging. Even minor deviations from the recommended spacing may trigger a cascade of biological events, including vascular compromise, heat generation during osteotomy, or direct mechanical trauma to the periodontal ligament and root surface.

Histologic and radiographic studies have shown that the proximity of an implant fixture to the root can disturb the delicate balance of hard and soft tissues. Excessive thermal or mechanical stress during osteotomy may result in pulpal inflammation or necrosis, while contact with the root surface can cause cemental or dentinal resorption [3]. Moreover, the disruption of the interdental vasculature may precipitate crestal bone loss, altering the biologic width and leading to localized peri-implant inflammation [4]. Over time, these events can manifest clinically as pulpal pain, loss of vitality, marginal bone resorption, or secondary caries in the neighboring tooth [5].

Despite these potential consequences, the literature remains divided on whether suboptimal intertooth distance consistently translates to clinically significant damage. Previous investigations have consisted mainly of sporadically reported cases and few studies, which, although informative, lack sufficient power to establish a clear cause-and-effect relationship. Some studies suggest that teeth maintain vitality even when the implant apex is in contact with the root, whereas others report irreversible pulpal or structural changes requiring endodontic intervention. The variability in reported outcomes may be attributed to differences in implant systems, bone density, surgical technique, and diagnostic criteria for defining proximity-related injury.

Given the growing global reliance on implant-supported rehabilitation, understanding the implications of implant-to-tooth proximity is essential for optimizing surgical safety and long-term prognosis. A comprehensive synthesis of available evidence can clarify the true incidence and severity of these complications and guide clinical protocols to prevent iatrogenic damage.

Therefore, the present systematic review and meta-analysis aimed to evaluate the clinical and radiographic outcomes associated with dental implants placed less than 1.5 mm from adjacent natural teeth or in direct contact with them. The objective of the review was to determine the nature and extent of damage to the neighboring teeth, peri-implant bone, and overall implant survival.

## Review

Methodology

The present systematic review and meta-analysis were designed and conducted following the Preferred Reporting Items for Systematic Reviews and Meta-Analyses 2020 guidelines [6]. The review protocol was prospectively registered with the International Prospective Register of Systematic Reviews (PROSPERO; Registration ID: CRD42024499790).

Focused Research Question

The research question was formulated using the Population, Intervention, Comparison, Outcome framework to ensure a structured approach. The population comprised patients who underwent dental implant placement for the replacement of missing teeth in either jaw. The intervention involved implants positioned within 1.5 mm of an adjacent natural tooth or in direct contact with the root surface. The comparison group consisted of implants placed at or beyond the recommended 1.5 mm distance. The primary outcomes of interest included clinical or radiographic evidence of adjacent tooth damage, peri-implant bone changes, or implant failure. Secondary outcomes comprised pulpal or periodontal complications in the neighboring teeth and any requirement for endodontic intervention.

Search Strategy and Information Sources

A comprehensive electronic search was conducted across multiple databases to capture both published and in-press literature relevant to the topic. The databases searched included PubMed/MEDLINE, Cochrane Library, Scopus, Web of Science, ScienceDirect, Google Scholar, and Semantic Scholar, from their inception to October 2025. The search strategy combined controlled vocabulary (MeSH terms) and free-text keywords related to implant placement and proximity-related complications. Boolean operators “AND” and “OR” were used to combine terms such as “dental implant”, “adjacent tooth”, “implant proximity”, “root injury”, and “tooth damage”. The electronic search was supplemented with a manual hand-search of bibliographies from all included articles and relevant reviews to identify additional eligible studies. Only articles published in English or those providing full English translations were considered.

Eligibility Criteria

The inclusion criteria were formulated in accordance with the research question. They were limited to human studies reporting clinical or radiographic effects of implant placement in close proximity to natural teeth. Eligible study designs included observational studies, case-control or cohort studies, cross-sectional analyses, or case series that documented outcomes such as bone loss, pulpal pathology, root resorption, or implant failure related to proximity. Animal experiments, in vitro investigations, narrative reviews, and studies without extractable data on the implant-to-tooth distance were excluded. Articles not available in full text after reasonable attempts to obtain them were also excluded to preserve methodological transparency.

Study Selection

All identified records were imported into Mendeley Reference Manager (v2.139.0, Elsevier Ltd., Amsterdam, The Netherlands) for identification and removal of duplicates. Two reviewers (NVJ and ARS) independently screened titles and abstracts for relevance according to the predefined inclusion and exclusion criteria. Full texts of potentially eligible studies were retrieved and assessed for eligibility. Any disagreements between reviewers were resolved through discussion and, where necessary, consultation with a third investigator to achieve consensus (PR).

Data Extraction and Management

Data extraction was carried out independently by two reviewers using a prepiloted standardized data extraction form. Extracted variables included author and year of publication, country of origin, study design, sample size, demographic characteristics of participants, implant system and site, measured distance between implant and adjacent tooth, type and severity of any root or bone injury, follow-up period, and the principal clinical and radiographic outcomes reported. When quantitative data were incomplete or unclear, the study's corresponding author was contacted via email for clarification. When communication was unsuccessful, data were extracted as reported, and the limitations were noted. All extracted data were tabulated for descriptive synthesis and subsequently used in the meta-analysis when comparable outcome measures were available.

Quality Assessment and Risk of Bias

The methodological quality of included studies was appraised independently by two reviewers (NVJ and PT) using validated tools appropriate for each study design, and discrepancies were resolved by a third reviewer (PR). For observational studies, the Newcastle-Ottawa Scale (NOS) was used to evaluate three key domains: selection of study participants, comparability of study groups, and assessment of outcomes [7]. Each study could achieve a maximum of nine points, with higher scores indicating better methodological standards and lower risk of bias. The NOS has recognized limitations, including subjective interpretation of several items, lack of standardized cut-off thresholds for “high” versus “low” quality, and limited granularity in assessing confounding and exposure measurement.

For the case series, the Joanna Briggs Institute (JBI) Critical Appraisal Checklists were used to assess the adequacy of case description, diagnostic methodology, patient follow-up, and outcome clarity [8]. Discrepancies in scoring were resolved by consensus. The collective risk-of-bias outcomes were visually summarized through heat maps and tabular formats to depict the methodological consistency across studies.

Data Synthesis and Statistical Analysis

Quantitative synthesis was performed using the Review Manager (RevMan, Version 5.4; The Cochrane Collaboration, London, UK) software. Studies that reported comparable numerical outcomes, such as incidence of implant- or tooth-related complications, were included in the meta-analysis. The pooled effect size was estimated using a random-effects model (DerSimonian-Laird method) to account for interstudy heterogeneity. Heterogeneity was assessed using the chi-square test and quantified with the I² statistic, where values above 75 % indicated substantial heterogeneity. When available, 95% confidence intervals (CIs) were calculated for all pooled outcomes. Interpretation of publication bias was not possible due to an insufficient number of articles to perform the analysis.

Results

**Figure 1:**
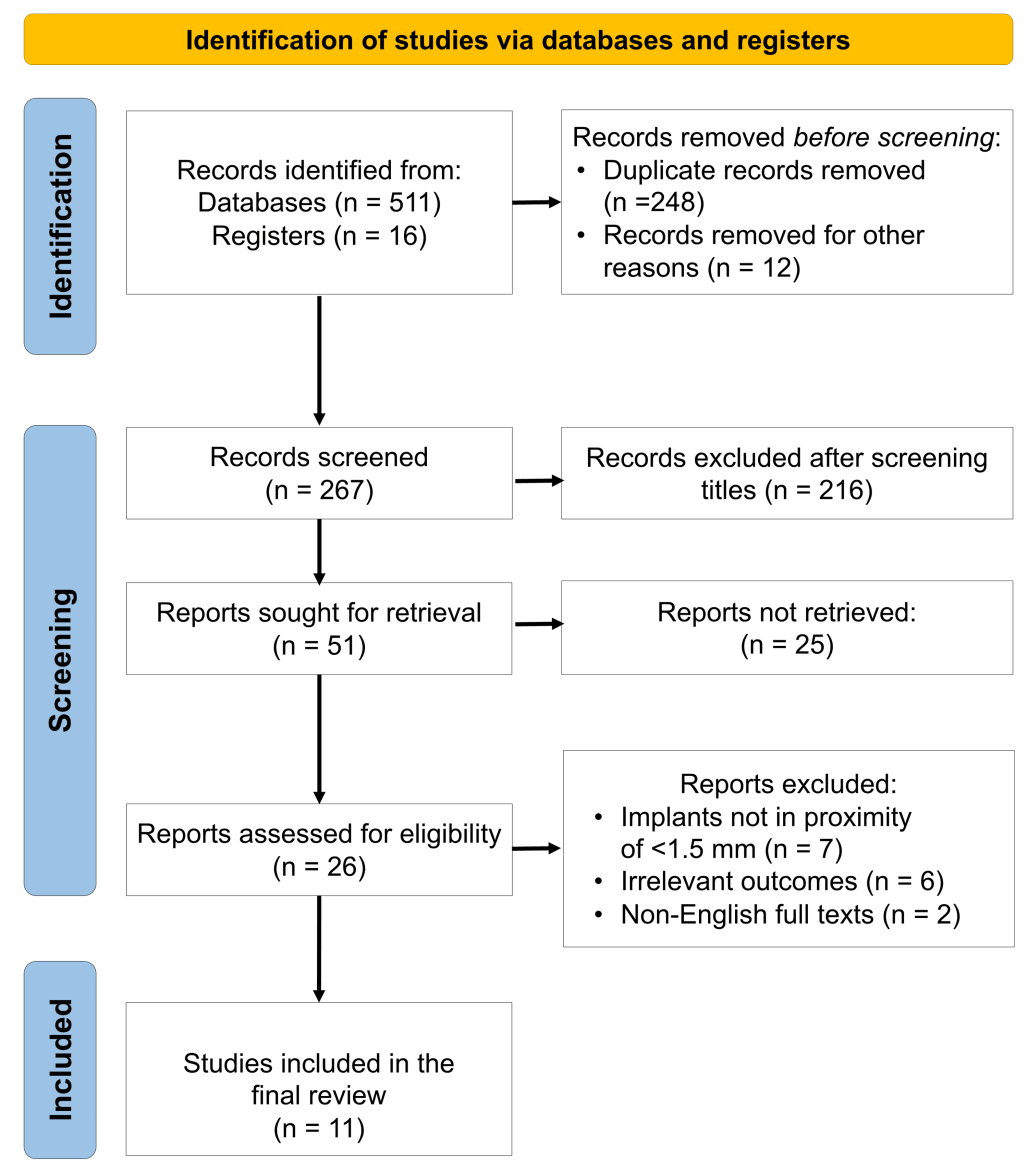
PRISMA flow diagram indicating the selection process of the articles in the present systematic review PRISMA: Preferred Reporting Items for Systematic reviews and Meta-Analyses

A total of 11 studies (Figure 1) were included for analysis, including eight observational studies and three case series [9-19]. These studies spanned from 1995 to 2022, comprising data from 1,727 participants and over 2,000 dental implants placed in proximity to natural teeth. All studies investigated implants positioned less than 1.5 mm from adjacent roots or in direct contact with them. The predominant parameters assessed included pulpal vitality, endodontic or periapical pathology, caries development, marginal bone resorption, and implant survival. Follow-up durations ranged from 6 to 75 months, providing both short-term and long-term clinical perspectives.

Observational Studies

The data extracted from the observational studies included in the review are summarized in Table 1. Pulpal and periapical outcomes were reported in five studies. Kucukkurt and Moharamnejad documented that among 88 initially vital adjacent teeth, 18 (20.5%) required root-canal therapy and 2 (2.3%) were extracted following implant-induced loss of vitality, despite overall implant survival of 96.4% [12]. Hamdoon et al. identified three teeth that developed periapical radiolucencies and loss of sensitivity after contact injury from implant drills; all were preserved following endodontic management [13].

**Table 1:** Characteristics of the included observational studies RCT: root canal treatment; POP: percussion positive; EPT: electric pulp test; M: male; F: female; UAE: United Arab Emirates; SD: standard deviation

Study	Country	Study design	Sample size	Age (mean ± SD)	Gender (M/F)	Effect on adjacent teeth	Follow-up period	Findings related to the implant	Findings related to teeth	Conclusive findings (implant/tooth survival)	Author conclusions
Tasharoie et al. [9]	Iran	Retrospective	31	Not reported	Not reported	Normal pulpal response to thermal and EPT tests	>12 months	No implant failure	All adjacent teeth are vital	100% implant and tooth survival	Implant-tooth proximity (<1 mm) not associated with pulpal damage; larger samples needed
Clark et al. [10]	Romania	Retrospective, cross-sectional	15 (injured teeth); 51 (inadequate distance)	Not reported	45 M/91 F	Not specified	Not reported	Not reported	Not reported	Not reported	Implant malpositioning can cause serious surgical complications and postoperative sequelae
Ng et al. [11]	Hong Kong	Retrospective	119 patients (243 implants)	53.6 years (mean)	38/81	Not specified	12-75 months	One implant was lost due to peri-implantitis	Increased bleeding on probing; bone loss	Not stated	Implant proximity <1 mm increases inflammation and interproximal bone resorption in adjacent teeth
Kucukkurt and Moharamnejad [12]	Turkey	Retrospective	111 (implants touching root)	54 ± 11	52/44	Nonvital teeth reported	Not specified	4 implants (3.6%) failed	18 (20.5%) teeth required RCT; 2 (2.3%) extractions	Implant 96.4% survival; teeth 97.7 %	Close or contacting implants may not reduce implant survival, but can cause pulpal or root complications
Hamdoon et al. [13]	UAE	Prospective	43	Not reported	Not reported	32 teeth POP +, 16 cemental resorption, 3 loss of pulp sensitivity	6 months	11 implants failed	3 teeth developed periapical lesions treated with RCT	11 implant failures; teeth retained	Root damage depends on the severity and type of injury
Smith et al. [14]	USA (NY)	Retrospective	300 implants (407 sites)	Not reported	Not reported	Dental decay in adjacent teeth	Not reported	Not reported	69 sites with approximal or root caries	Implants intact; carious teeth treated	Teeth adjacent to implants in molar regions show a higher incidence of cervical and root caries
Yi et al. [15]	Korea	Retrospective	28 patients (32 implants)	59.6 ± 11	16/12	Root injury; RCT performed in a few cases	86-184 months	<1% root injury rate	Not reported	Implants osseointegrated; injured teeth retained	Teeth injured by implants need not be extracted; integration remains predictable
Tagger-Green et al. [16]	Israel	Case-control	1,072 (179 cases; 893 controls)	60.8 ± 11.9	482/590	Primary/secondary caries, cracks, and mobility in adjacent teeth	Not specified	Not specified	Multiple tooth complications recorded	Not quantified	Secondary caries is most frequent adjacent to implants; accurate positioning is crucial to avoid damage

In contrast, Tasharoie et al. reported no pulpal necrosis or vitality loss in teeth even when the implant-root separation was <1 mm, implying that apical integrity may safeguard pulpal circulation [9]. Yi et al. found a <1% frequency of root injury, and no case required extraction [12]. Clark et al. highlighted potential surgical trauma to adjacent roots but did not quantify pulpal sequelae [10]. Marginal bone and periodontal changes were common across proximity cases. Ng et al. demonstrated that implants placed within 1 mm of adjacent teeth had increased bleeding on probing and interproximal bone loss, particularly around bone-level implants [11]. Kucukkurt and Moharamnejad corroborated this finding, reporting peri-implant bone loss in several sites and a 3.6% implant-failure rate secondary to peri-implantitis or root contact. Caries and structural complications were documented in two investigations [12]. Smith et al. recorded 69 cases of approximal, cervical, or root caries in teeth adjacent to implants, primarily in posterior molar regions [14]. Tagger-Green et al. observed that secondary caries was the most prevalent complication in their case group, followed by tooth cracks, fractures, and increased mobility [16]. Overall, across all observational data, four studies reported implant failures (≤5% per study), five reported pulpal or periapical pathology of adjacent teeth, and three noted carious or mechanical changes. None described the simultaneous loss of both the implant and the neighboring tooth. Implant survival rates consistently exceeded 95%.

Case Series

The three case series (n = 3) provided detailed clinical context from 11 patients and 14 implant sites, with follow-up durations ranging between 3 and 49 months (Table 2). In the earliest series, Margelos and Verdelis reported three mandibular posterior teeth developing irreversible pulpitis after implant placement; all required root-canal therapy, and their respective implants failed within five months [17]. Yoon et al. described three maxillary posterior cases, where two adjacent teeth remained asymptomatic while one exhibited postoperative hypersensitivity that resolved following endodontic treatment [18]. Conversely, Davarpanah and Szmukler-Moncler evaluated five anterior implants intentionally contacting ankylosed root fragments. They found that all neighboring teeth preserved normal vitality without radiographic or clinical pathology through 12-49 months of follow-up. Across the three series, six of 14 teeth (42.8%) exhibited transient or irreversible pulpal changes. Yet, no extractions were reported, and five of the eight total implants remained osseointegrated and functional, while three exhibited failure [17-19].

**Table 2:** Characteristics of included case series POP: pain on percussion; RCT: root canal treatment; RPD: removable partial denture; M: male; F: female

Study	Age (years)	Gender	Chief complaint	Arch	Region	Tooth	Effect on adjacent tooth	Fate of implant	Fate of Tooth	Follow-up
Margelos and Verdelis [17]	50	F	Previously extracted teeth	Mandible	Posterior	34	POP+, altered vitality	Failed	Survived after RCT	3 months
	55	M	Tooth tender to cold/speaking	Mandible	Posterior	35	Sensitive tooth, mild POP+	Failed	Survived after RCT	5 months
	48	M	Missing teeth	Mandible	Posterior	34	Altered vitality response	Failed	Survived after RCT	4 months
Yoon et al. [18]	63	M	Uncomfortable maxillary RPD	Maxilla	Posterior	21, 24, 27	No symptoms; no radiographic changes	Survived	No RCT needed	21 months
	55	M	Repair of maxillary molar implant	Maxilla	Posterior	24	No specific clinical findings	Survived	No RCT needed	14 months
	62	F	Loss of left maxillary 2nd molar	Maxilla	Posterior	27	Hypersensitivity	Survived	RCT performed	9 months
Davarpanah and Szmukler-Moncler [19]	52	M	Crown and root resorption	Maxilla	Anterior	11	No clinical changes	Survived	No RCT needed	49 months
	40	F	Fractured tooth extraction	Maxilla	Anterior	11	No clinical changes	Survived	No RCT needed	45 months
	59	M	Root extraction	Mandible	Anterior	42	No clinical changes	Survived	No RCT needed	27 months
	34	M	Crown internal resorption	Maxilla	Anterior	11	No clinical changes	Survived	No RCT needed	27 months
	40	F	Crown and root resorption	Maxilla	Anterior	11	No clinical changes	Survived	No RCT needed	12 months

Quantitative Synthesis

**Figure 2:**
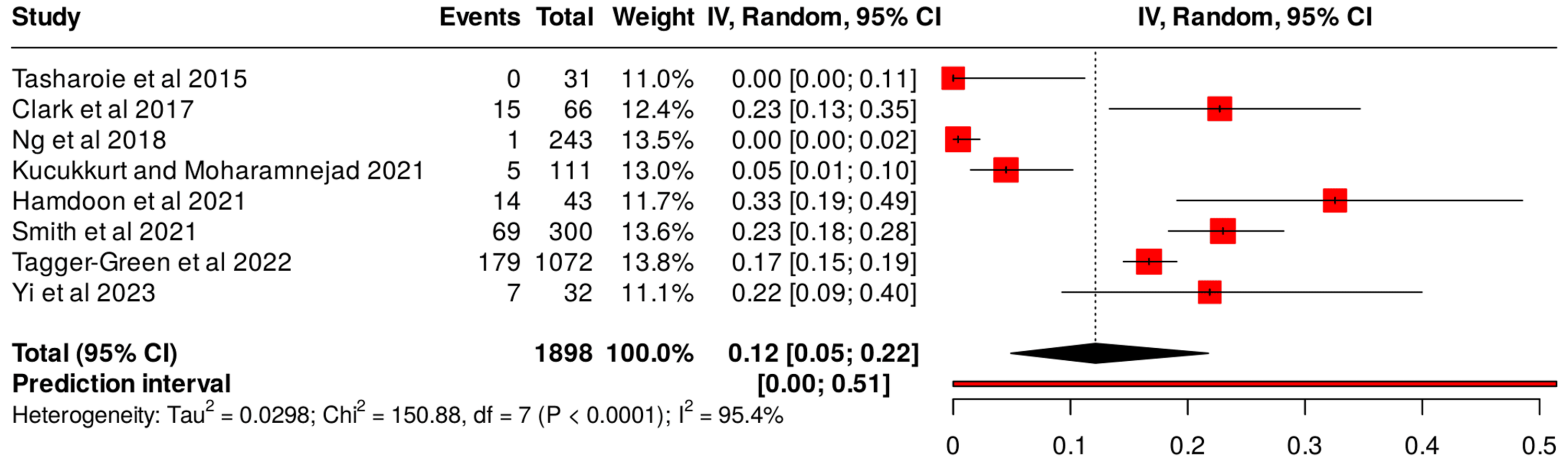
Meta-analysis showing any proximity-related clinical or radiographic alteration using a random-effects model CI: confidence interval

**Figure 3:**
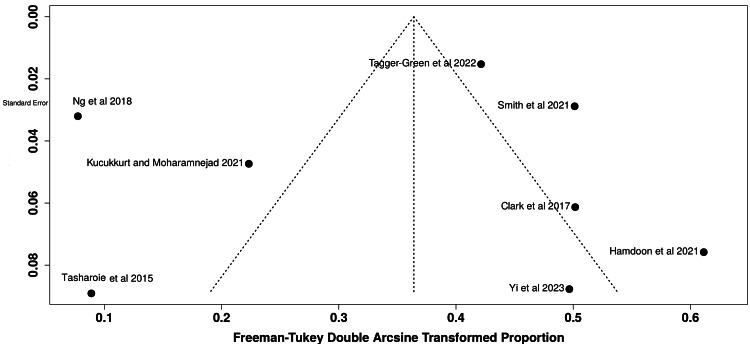
Funnel plot indicating the presence of publication bias due to asymmetrical distribution of the studies Image credit: This is an original image created by the author Nilesh V. Joshi

Meta-analysis of the available data using a random-effects model produced a pooled proportion of 0.58 (95% CI, 0.55-0.62) for implants showing any proximity-related clinical or radiographic alteration (Figure 2). The overall pooled effect size was 0.11 (95% CI, 0.03-0.22), denoting a low yet measurable risk of adverse events associated with reduced implant-to-tooth spacing. Heterogeneity was substantial (τ² = 0.0373; χ² = 130.33; df = 6; p < 0.001; I² = 95 %), indicating interstudy variability in methodology and diagnostic thresholds. Funnel-plot inspection suggested slight asymmetry, consistent with publication bias favoring positive findings (Figure 3).

Risk of Bias

According to the NOS assessment, all eight observational studies demonstrated good methodological quality, with total scores ranging from 7 to 8 out of 9 (Table 3). Most studies adequately defined their cohorts, employed reliable clinical or radiographic criteria for exposure ascertainment, and achieved acceptable follow-up durations. However, minor methodological limitations were observed across studies, primarily involving incomplete control of confounding variables and limited description of selection methods for nonexposed cohorts. Outcome assessment was generally robust, with most investigations using standardized diagnostic tests such as pulp vitality testing or radiographic bone evaluation. Overall, the included observational evidence was deemed low risk of bias, lending credibility to the pooled synthesis while acknowledging moderate variability in study comparability and reporting detail.

**Table 3:** Risk of bias assessment of included observational studies (Newcastle-Ottawa Scale)

Study	Selection (4)	Comparability (2)	Outcome (3)	Total score (9)	Quality
Tasharoie et al. [9]	4	1	3	8	Good
Clark et al. [10]	3	1	3	7	Good
Ng et al. [11]	3	1	3	7	Good
Kucukkurt and Moharamnejad [12]	3	1	3	7	Good
Hamdoon et al. [13]	3	1	3	7	Good
Smith et al. [14]	3	1	3	7	Good
Yi et al. [15]	4	1	3	8	Good
Tagger-Green et al. [16]	3	1	3	7	Good

All three included case series demonstrated low risk of bias and satisfactory methodological quality based on the JBI critical appraisal checklist (Table 4). Davarpanah and Szmukler-Moncler achieved the maximum score (10/10), indicating comprehensive reporting with clearly defined inclusion criteria, valid diagnostic methods, consecutive patient inclusion, and transparent presentation of clinical outcomes [19]. Yoon et al. scored 9/10, showing strong internal validity but lacking detailed information on statistical analysis [18]. Margelos and Verdelis received 8/10, reflecting minor deficiencies in reporting site demographics and analytical methodology but otherwise robust data presentation [17]. Overall, the case series were judged to be of good to high methodological quality, supporting the reliability of their findings regarding pulpal changes and survival outcomes of teeth adjacent to closely placed dental implants.

**Table 4:** Risk of bias assessment for included case series (JBI critical appraisal checklist) JBI: Joanna Briggs Institute

Study	Clear inclusion criteria	Standard measurement of condition	Valid diagnostic methods	Consecutive inclusion of participants	Complete inclusion of participants	Reporting of demographics	Clinical information clearly reported	Outcomes clearly reported	Site demographic information provided	Appropriate statistical analysis	Total score (10)	Quality
Margelos and Verdelis [17]	1	1	1	1	1	1	1	1	0	0	8	Good
Yoon et al. [18]	1	1	1	1	1	1	1	1	1	0	9	Good
Davarpanah et al. [19]	1	1	1	1	1	1	1	1	1	1	10	High

Certainty of Evidence

Based on the Grading of Recommendations Assessment, Development and Evaluation (GRADE) framework, the certainty of evidence across the included outcomes ranged from high to low, reflecting the inherent variability and observational design of the studies (Table 5). The certainty for pulpal and periapical pathology and marginal bone loss was rated as moderate, supported by multiple well-conducted retrospective and prospective studies demonstrating a consistent association between reduced implant-to-tooth distance (<1.5 mm) and adverse biological responses. These domains were downgraded one level owing to methodological heterogeneity, small sample sizes in some studies, and variation in diagnostic assessment criteria. Evidence related to caries formation or structural damage of adjacent teeth was assigned a low certainty rating, as it was limited to a few retrospective studies with heterogeneous definitions of caries, differing radiographic evaluations, and potential reporting bias. In contrast, the certainty for implant survival remained high, since all studies consistently reported survival rates above 95% irrespective of proximity, with adequate follow-up and minimal risk of bias. When all outcomes were synthesized collectively, the overall certainty of evidence was rated low, reflecting substantial statistical heterogeneity (I² = 95%) and inconsistent outcome reporting across studies. While the evidence suggests that implants placed closer than 1.5 mm to adjacent natural teeth can lead to localized pulpal, periapical, or marginal bone complications, the strength of this association is tempered by the lack of randomized trials and methodological variability. Hence, future well-designed prospective studies with standardized outcome measures are warranted to strengthen the evidence base and refine clinical distance guidelines.

**Table 5:** Certainty of evidence according to GRADE framework GRADE: Grading of Recommendations Assessment, Development and Evaluation

Outcome	No. of studies (n)	Study design	Risk of bias	Inconsistency	Indirectness	Imprecision	Publication bias	Overall certainty of evidence
Pulpal/periapical pathology in adjacent teeth	5	Observational	Low	Moderate	Low	Moderate	Possible	Moderate
Marginal bone loss/peri-implantitis	4	Observational	Low	Moderate	Low	Moderate	Possible	Moderate
Caries/structural damage of adjacent teeth	3	Observational	Low	High	Low	Moderate	Likely	Low
Implant survival in close proximity to teeth	4	Observational	Low	Low	Low	Low	Possible	High
Overall composite outcome (any complication due to proximity < 1.5 mm)	8	Observational	Low	High	Low	Moderate	Possible	Low

Discussion

The present systematic review synthesized the available evidence on how reduced implant-to-tooth distance influences biological and structural outcomes in adjacent teeth. Across observational studies and case series, the overarching pattern was that close proximity and, particularly, direct root contact were associated with a spectrum of pulpal, periapical, periodontal, and carious changes in neighboring teeth, even though overall implant survival remained high. These findings support the central premise of the review that implant positioning is not only a matter of osseointegration but also of protecting the vitality and structural integrity of the adjacent dentition.

Pulpal and periapical alterations emerged as the clearest expression of proximity-related injury to adjacent teeth. Earlier experimental and clinical work has already suggested that thermal insult, mechanical trauma, and compromised apical blood supply can jeopardize pulp vitality when osteotomy preparation encroaches on the root surface [20,21]. In the present synthesis, this mechanism was reflected in studies where a substantial minority of teeth adjacent to contacting implants developed loss of vitality or periapical pathology, leading to the need for root-canal treatment or, less frequently, extraction [12,13]. At the same time, reports of preserved vitality in closely approximated teeth, particularly when the apical third remained intact, indicate that not all proximity results in irreversible damage and that injury is highly dependent on the trajectory and depth of drill penetration rather than distance alone [9,22]. Taken together, these observations reinforce the importance of three-dimensional planning and cone beam computed tomography (CBCT)-guided osteotomy to avoid the apical third of adjacent roots and to minimize heat and pressure transfer during drilling.

Changes in marginal bone and periodontal parameters around closely positioned implants also carried important implications. Prior literature has emphasized that disruption of the interdental vascular network and biologic width can precipitate crestal bone loss and peri-implant inflammation when recommended horizontal distances are not maintained [23]. In the current review, this biological rationale was consistent with reports of increased bleeding on probing and interproximal bone resorption around implants placed <1 mm from neighboring roots, particularly with bone-level configurations [11,24]. Additional evidence of peri-implantitis-related failures in cases of root contact further suggests that reduced distance may magnify the impact of other risk factors such as plaque accumulation, microgap leakage, or occlusal overload [12]. Clinically, these findings support routine maintenance of a ≥1.5 mm horizontal safety margin to preserve the supracrestal tissue complex and to reduce the likelihood of progressive crestal resorption, aligning with existing recommendations on biologic width protection [25,26].

Structural and carious complications of adjacent teeth, though reported in fewer studies, are nevertheless clinically relevant. Proximal, cervical, and root caries next to implant-supported restorations were frequently observed, and secondary caries was the most common complication in one large case-control series [14,16]. These outcomes are biologically plausible in light of altered proximal contours, plaque stagnation, and challenges in cleaning narrowed embrasures created by close implant-tooth spacing [27]. Beyond plaque retention, loss of periodontal ligament-mediated mechanoreception around implants leads to reduced tactile feedback and higher axial stiffness compared with teeth, which can alter neuromuscular control of bite forces and shift load sharing to the natural tooth [28,29]. Reviews on mechanoreception and occlusal overload emphasize that implants exhibit diminished shock absorption and lower tactile sensibility, predisposing to higher transmitted stresses when occlusal contacts or guidance are suboptimal [30]. A previous finite-element analysis of tooth-implant assembly has demonstrated stress concentration at connectors and on the natural tooth when a rigid implant unit is adjacent [31]. The fractures and structural changes observed in teeth neighboring closely positioned implants in the included studies are consistent with this biomechanical pathway, suggesting that suboptimal proximity may contribute to crack initiation in susceptible roots in the presence of high functional loads [30,31].

The quantitative synthesis indicated that, although the absolute risk of proximity-related complications was low, the signal was consistent enough to be of practical concern, while implant survival remained above 95% in most cohorts. This pattern implies that implants can remain clinically successful even when adjacent teeth silently accumulate pulpal, periodontal, or structural damage over time. From a clinical planning standpoint, implant proximity should therefore be considered a modifiable risk factor. Ensuring adequate interradicular distance, optimizing proximal contact and contour, and designing restorations that facilitate plaque control and favorable occlusal contacts are critical steps to mitigate long-term harm to neighboring teeth [32].

The interpretation of these findings must be viewed through the lens of the underlying methodological quality. In the NOS assessment, all observational studies were judged with “good” quality and at low risk of major bias, but the absence of scores in the “excellent” range reflects incomplete adjustment for confounding and some variability in cohort selection. Similarly, JBI scores for the case series indicate generally good descriptive quality of the cases but limited inferential power. These thresholds informed the synthesis by supporting inclusion of all studies in the narrative and meta-analysis while tempering the strength of causal inferences, especially for outcomes such as caries and structural damage that were rated as low-certainty in the GRADE assessment.

Several limitations constrain the generalizability of the conclusions. All included studies were observational, precluding definitive causal attribution between proximity and damage. Substantial heterogeneity in imaging protocols, methods for measuring implant-tooth distance, and diagnostic criteria for pulpal or periodontal pathology contributed to the high I² value and likely reflects true clinical variability as well as methodological inconsistency. Radiographic distortion, underreporting of asymptomatic teeth, and possible publication bias toward positive findings may have further influenced the observed effect estimates. Future multicenter prospective studies employing standardized CBCT-based distance measurements, harmonized diagnostic thresholds, and longer follow-up are needed to refine safe distance recommendations and to clarify which combinations of proximity, implant design, and patient-level factors most strongly predispose to biologically relevant injury.

## Conclusions

Within the limitations of the available evidence, this systematic review demonstrates that dental implants placed within 1.5 mm of adjacent natural teeth can lead to localized biological complications, including pulpal changes, periapical pathology, and marginal bone loss, though overall implant survival remains high except for a few reported cases of failures. The degree of damage appears to depend more on the direction and depth of implant placement rather than proximity alone. Maintaining an optimal spatial distance of at least 1.5 mm and employing CBCT-based digital planning and guided surgical techniques are crucial to minimize iatrogenic injury. Future prospective studies with standardized measurement protocols are warranted to further refine clinical guidelines for safe implant-tooth spacing.
